# A national multiple baseline cohort study of mental health conditions in early adolescence and subsequent educational outcomes in New Zealand

**DOI:** 10.1038/s41598-023-38131-8

**Published:** 2023-07-07

**Authors:** Emma Gorman, Nicholas Bowden, Jesse Kokaua, Brigid McNeill, Philip J. Schluter

**Affiliations:** 1 School of Organisations, Economy and Society, University of Westminster, 35 Marylebone Rd, Marylebone, London, NW1 5LS UK; 2 Department of Women’s and Children’s Health, University of Otago, 201 Great King St, Dunedin, 9016 New Zealand; 3 A Better Start National Science Challenge, Auckland, New Zealand; 4 Va’a O Tautai-Centre for Pacific Health, Division of Health Sciences, University of Otago, 78 Frederick St, Dunedin, 9017 New Zealand; 5 Te Kāhui Pā Harakeke, Child Well‑being Research Institute, Te Whare Wānanga o Waitaha, University of Canterbury, Christchurch, 8041 New Zealand; 6 Te Kura Whakangungu Kaiako, The School of Teacher Education, Te Whare Wānanga o Waitaha, University of Canterbury, Christchurch, 8041 New Zealand; 7 Te Kaupeka Oranga, Faculty of Health, Te Whare Wānanga o Waitaha, University of Canterbury, Christchurch, 8041 New Zealand; 8 Primary Care Clinical Unit, School of Clinical Medicine, The University of Queensland, Brisbane, Australia

**Keywords:** Public health, Psychiatric disorders

## Abstract

Young people experiencing mental health conditions are vulnerable to poorer educational outcomes for many reasons, including: social exclusion, stigma, and limited in-school support. Using a near-complete New Zealand population administrative database, this prospective cohort study aimed to quantify differences in educational attainment (at ages 15–16 years) and school suspensions (over ages 13–16 years), between those with and without a prior mental health condition. The data included five student cohorts, each starting secondary school from 2013 to 2017 respectively (N = 272,901). Both internalising and externalising mental health conditions were examined. Overall, 6.8% had a mental health condition. Using adjusted modified Poisson regression analyses, those with prior mental health conditions exhibited lower rates of attainment (IRR 0.87, 95% CI 0.86–0.88) and higher rates of school suspensions (IRR 1.63, 95% CI 1.57–1.70) by age 15–16 years. Associations were stronger among those exhibiting behavioural conditions, compared to emotional conditions, in line with previous literature. These findings highlight the importance of support for young people experiencing mental health conditions at this crucial juncture in their educational pathway. While mental health conditions increase the likelihood of poorer educational outcomes, deleterious outcomes were not a necessary sequalae. In this study, most participants with mental health conditions had successful educational outcomes.

## Introduction

Mental health conditions are a leading contributor to the global burden of disease^1,2^, with onset of over half of all cases occurring before age 14 years^3^. The global prevalence of mental health conditions is increasing in recent cohorts of young people, particularly internalising conditions such as depression and anxiety^4,5^. New Zealand is no exception, with self-reported measures of psychological distress increasing over the past decade^6,7^. This trend is likely the consequence of multiple factors, including the COVID-19 pandemic and increasing social media use^8^. Young people experiencing mental health conditions can be vulnerable to social exclusion, stigma and educational difficulties—the consequences of which can extend into adulthood^9–11^.

Educational attainment is a key predictor of subsequent health, wellbeing and economic outcomes for individuals^12–16^, as well as contributing to economic growth and national living standards^17^. Improving educational attainment is a policy priority in most countries. While New Zealand has historically had high performance on international standardised assessments, such as the Organisation for Economic Co-operation and Development (OECD) Programme for International Student Assessment (PISA), recent decades have seen it slide down the international rankings^18,19^. Similarly, the 2017 report on Progress in International Literacy Study (PIRLS) showed New Zealand literacy rates for 10 year-old children have declined over the preceding 5 year period^20^. Further, the significant literacy learning gap between certain groups (i.e. between students of different ethnicities, gender or socioeconomic backgrounds)—reportedly as large as four years of schooling—is not decreasing^21^. A range of factors are hypothesised to underpin this decline including growing societal inequities, lack of uptake of evidence-based teaching practices in early literacy instruction, change in qualification system in upper secondary, or the negative impact of digital technologies^22^. Improving educational performance is high on New Zealand’s policy agenda, indicated by the recent release of the Literacy, Communication and Maths Strategy^23^ and proposed changes around literacy and numeracy requirements within secondary schooling.

Associations between lower mental health and educational attainment have been widely explored and documented^9,24–29^. With some exceptions^29^, there is a negative association between mental health condition and attainment. Concerns about mental health, and its associations with educational outcomes, have become more salient during the novel coronavirus disease 2019 (COVID-19) pandemic, as many children faced severe disruptions to home and school life. Initial data suggest COVID-19 and its sequelae have exacerbated existing inequalities in educational experiences and mental health^30,31^, and lead to increases in externalising conditions post-pandemic in adolescents with pre-existing behavioural disorders^32^. Links between mental health and educational outcomes may occur through several potential mechanisms, including stigma attached to disruptive behaviour and a lack of appropriate in-school support. Poor mental health may also exert a direct effect on education outcomes, through time out of school, reduced ability to concentrate, lower motivation or confidence. However, educational outcomes vary among young people with mental health conditions, and poorer outcomes are not a necessary corollary. Moreover, the extant literature largely relies on mental health measures derived from survey data^25^. Such measures may be subject to differential measurement error^33^, and health-related selective nonresponse^34^, with potential to bias estimations of prevalence and association if mismeasurement or misclassification is related to the quantities of interest. This issue of survey non-response is likely to be especially pronounced for populations often under-served by the research community, such as those experiencing more severe mental health conditions^35^.

New Zealand’s Integrated Data Infrastructure (NZIDI) is a large repository of linked administrative and survey data established in 2011, bringing together national data sourced from a range of government and non-government agencies^36^. Within New Zealand, it links prospectively captured data for a near-complete national population on a range of variables including mental health conditions, educational outcomes, and sociodemographic characteristics. The prospective nature of the data affords enhanced credibility of findings compared to retrospective cross-sectional studies which are often employed in this literature. The NZIDI's large sample size permits high statistical power, and robust analyses of small subgroups and less frequent events—which are not always feasible in sample-surveys. A novel methodology specifically designed to identify mental health conditions among children and young people, combining clinical judgement with routinely-collected service mental health care usage records, has been developed and successfully applied to the NZIDI data^37^. These data and methods redress the methodology and coverage issues common to surveys investigating adolescent mental health. Access to the data is provided by Statistics New Zealand, under conditions designed to give effect to the provisions of the Statistics Act 1975^38^, which ensures appropriate confidentiality of participants.

Educational attainment or success for adolescents can be characterised in myriad ways over multiple domains. Two sentinel measures with good face validity are qualification attainment and school inclusion/exclusion. Within New Zealand, the first nationally externally assessed educational qualification occurs in school Year 11 when pupils are normally aged 15–16 years. Known as the National Certificate of Education Attainment (NCEA), the Level 1 Certificate is an important gateway to continued education, employment and training. However, there remain substantial sociodemographic disparities in attainment, with boys, Māori and Pacific peoples, and those attending schools located in less affluent areas, having lower levels of attainment^39,40^. Conversely, school exclusions (stand-downs and suspensions) are a negative education outcome. In New Zealand, stand-downs are student removals from school for no more than five days in a school term or a total of ten days in a school year (the stand-down rate in 2021 was 26.8 per 1000 students), whereas suspensions are a longer removal from school until further action is taken (3.1 per 1000 students in 2021)^41^. Students can be suspended due to continual disobedience which is harmful or dangerous to other students, gross misconduct which is harmful or dangerous to other students, or behaviour that risks serious harm if the student is not suspended^42^. Stand-down and suspension results in time out of school, social stigma, and disruption to routine; therefore representing a potential precursor to reduced attainment in examinations and poorer later life outcomes^43^. Exclusion policies and practices may be amenable to modifications to reduce impacts on learning and attainment, and mitigate links between experience of mental health conditions and subsequent poorer attainment. Both NCEA Level 1 results and exclusion data appear within the NZIDI.

Drawing on the NZIDI, a national integrated database, this study has two primary aims; namely, to quantify differences in (i) qualification attainment at school Year 11 (normally aged 15–16 years), and (ii) stand-downs or suspensions from school Years 9–11 (normally aged 13–16 years), between those with and without a prior mental health condition, after adjusting for a range of socio-economic and demographic characteristics.

## Methods

### Study design

The analyses are based on a prospective cohort study design. Five successive cohorts of Year 9 (aged 12–13 years; mean age 12.8 years) secondary students with baseline measurement in 2013 (n = 57,582), 2014 (n = 53,406), 2015 (n = 53,304), 2016 (n = 53,601) and 2017 (n = 55,008), respectively, were established. Each cohort was followed for three years.

### Data

The data used in this study is NZIDI, a repository of administrative and survey data which links records at the individual level from national data sources across a range of government and non-government agencies^36^. The NZIDI captures records of individuals’ interactions with government agencies for health, social services, education, justice, geography, housing and economics (tax and income). Data from the 2013 Census are also included. The data sources in the NZIDI, such as routinely collected records from government agencies, are linked via established record-linkage methodologies. The primary goal of record-linkage is to link an entity (e.g., a person) from one file to the same entity in other file(s)^44,45^. The aim of the NZIDI methodology is to achieve a high linkage rate between data sources, while maintaining a false positive rate below 2%^46^. This is possible due to the granular nature of the variables which are used in the linkage. The core variables used for linking datasets are: First name, last name, sex, year of birth, month of birth, day of birth, which are supplemented by additional identifying variables as available in a given dataset. Access and use of the data are governed by SNZ ‘Five Safes’ approach statistical disclosure control^47^.

### Study population

The base study population comprises an Ever Resident Population (ERP), which defines the resident population each year in the NZIDI^48^. For each year, individuals are included in the ERP if they have engaged with key services (e.g., tax, healthcare, education) in New Zealand during the two years preceding. Individuals are excluded if they have died or emigrated to another country before the end of that year. Moreover, individuals were excluded from the study population if they resided in another country for more than six months during the two year window for identifying mental health conditions (see the mental health measure sub-section) or more than one year outside of New Zealand during the education outcome window (see education measures sub-section) because in these cases we were unable to reliably measure these variables. Here, five successive cohorts of secondary school students were defined and extracted: those who entered their first year of secondary school (school Year 9, normally aged 13–14 years) in years 2013, 2014, 2015, 2016 or 2017. These years were selected to ensure consistent constituent data source coverage for the mental health condition identification^37^.

### Primary educational measures

The National Certificates of Educational Achievement (NCEA) are national qualifications for secondary school students in New Zealand, governed by the New Zealand Qualifications Authority. NCEA is recognised by employers, and used for selection by universities and vocational higher education. Students can choose a broad range of subjects, with Maths, English and Science being compulsory. In each subject, skills and knowledge are assessed against a range of standards. Once a standard is obtained, the student gains credits. To achieve NCEA Level 1, 80 credits are required. NCEA Level 1 is undertaken in year 11, aged 15–16 years, and is an important stepping-stone to further employment or study. Two binary educational outcome measures were defined for each student: whether the NCEA Level 1 Certificate qualification at Year 11 (normally aged 15–16 years) was gained or not; and whether any stand-downs or suspensions over school Years 9–11 (normally aged 13–16 years) were recorded or not. School Year 11 is the final year of compulsory secondary schooling in New Zealand.

### Primary mental health measures

Mental health conditions were created using an established, published, case identification methodology^37^. The full details of the methodology are available in the relevant publication^37^, and we provide an outline of the salient features for the present paper as follows. In brief, this approach brings together records from multiple data sources within the NZIDI, in conjunction with clinical judgement, to identify and classify clinically relevant mental health conditions among children and young people. Data sources include the following Ministry of Health national collections: the Programme for the integration of mental health data (PRIMHD), a national collection of publicly funded specialist mental health service use (service-use and diagnoses); the National Minimum Dataset (NMDS), a national collection of publicly funded hospital admissions (service use and diagnoses); Socrates, a national database of the Ministry of Health’s Disability Support Service clients and service providers; and, the Pharmaceutical collection, claims and payment information from pharmacists for government-subsided medication dispensing. The authors of this case identification method employed a two-staged approach to classify 13 mental health conditions. First, a shortlist of thirteen mental health conditions was derived by a team of clinical experts (anxiety, depression, emotional problems (indeterminant anxiety or depression), bipolar disorders, substance problems, eating problems, disruptive behaviours, psychosis, personality disorders, sleep problems, self-harm, other mental health problems, and mental health not defined). In the second stage, a panel of eight specialists, including a clinical psychologist, four child and adolescent psychiatrists, and three researchers in child and adolescent mental health, independently assigned a combination of diagnostic codes (ICD-10-AM and DSM-4 codes from PRIMHD, ICD-10-AM codes from NMDS, and assigned diagnosis codes upon referral from Socrates), service-use activity (PRIMHD) and inferences from medication prescribing (Pharmaceutical collection) to classify mental health conditions (see supplementary material for details). Disagreement of code assignment was resolved through discussion and consensus. The case identification method was not formally validated. The codes lists for each grouping are reported in Supplementary Materials Table A1.

For the present study, we created three exposure variables, identifying mental health conditions from records during the 2 years prior (approximately aged 11–13 years) to starting secondary school (to reduce the possibility of reverse causality). The first variable, ‘emotional conditions’, consisted of anxiety, depression, and indeterminant anxiety or depression. The second, termed ‘behavioural conditions’, comprised attention-deficit/hyperactivity disorder (ADHD), conduct problems, and oppositional defiant disorder (denoted as disruptive behaviours in the original case identification method). The final variable was a composite indicator of any mental health condition and included emotional and behavioural conditions, in addition to the smaller subgroups of psychosis, substance problems, sleep problems, eating disorders, self-harm, other mental health, and mental health not defined (bipolar disorders and personality disorders were excluded as these pertain to young people older than those included in the present study). The three exposure variables are not mutually exclusive so individuals may be identified with multiple mental health exposure groups.

### Sociodemographic and potentially confounding variables

Sociodemographic variables measured at baseline included child’s sex (male, female), age-at-school-entry (years), and ethnicity. These measures are sourced from multiple collections including Census data, birth records, health, and education data. The measures are generally self-reported. Here ethnicity comprised five groupings: Māori, Pacific peoples, Asian, Middle Eastern/Latin American/African (MELAA), and European/Other. This variable was classified using the total response approach, whereby categories are not mutually exclusive and individuals can belong to multiple ethnic groups. Māori represent the descendants of indigenous inhabitants of New Zealand, while the other groups refer to migrants, or descendants of original migrants, from the regions after which they are named. Additionally, highest level of parental education attainment, household income, level of deprivation, and residential location variables were used. Taken from the 2013 Census, highest level of parental education attainment (from either parent) was categorised into five groups: not reported, no qualification, secondary school qualification, post-secondary school qualification, and university level. Household income (NZD), also drawn from the 2013 Census, was banded into the following categories ≤ $25,000; $25,001–$50,000; $50,001–$100,000; $100,101–$150,000; ≥ $150,001 (median household income was $63,800 in 2013)^49^. Deprivation was measured using the New Zealand Deprivation Index (NZDep)^50^. This small-area (around 30 households) deprivation measure combines Census information on income, employment, qualifications, communication, support, living space, transport and home ownership into a single continuous measure. This was partitioned into quintiles, with one representing the least deprived households and five representing those with the greatest level of deprivation. Finally, residential location used Statistics New Zealand (SNZ) 2016 urban/rural classification has five categories: (1) main urban (population of at least 30,000), (2) secondary urban (population 10,000–29,999), (3) minor urban (population 1000–9999), (4) rural centre (population 300–999) and (5) other rural (population < 300). These were collapsed into two groups to create a binary indicator: urban (main urban, secondary urban and minor urban area) and rural (rural centre and other rural).

### Statistical analyses

The reporting in this study follows the Reporting of studies Conducted using Observational Routinely-collected Data (RECORD) guidelines^51^. After describing the participant flow and their characteristics, complete-case multilevel mixed-effect modified Poisson regressions were used to relate mental health indication to educational outcomes, namely NCEA Level 1 Certificate achievement and stand-down or suspension indication^52^. Modified Poisson regressions, with robust variance estimators, were chosen to provide direct estimates of incidence rate ratio’s (IRR’s) because the rates of educational outcome were not rare^53^, and conventional logistic regression models produce odds ratios which are inflated estimates of IRRs. School-level random effects were included to account for intra-school correlation. Three model specifications were investigated, each sequentially adding a new set of potential confounders. Model 0 reported unadjusted IRR’s, which gives the risk of experiencing an educational outcome if a student has a prior mental health condition compared to students with no prior mental health condition, adjusting only for school cohort year. Model 1 reported adjusted IRRs by adding participants’ demographic variables (namely: sex, age-at-school-entry and ethnicity); Model 2 further adds potential socioeconomic confounders (namely: highest parental education level, household income bands, quintiles of area-deprivation and residential location). Because previous studies have found differential associations between mental health conditions and education-related outcomes by sex, we estimated all models separately by sex^54^, with results reported in Supplementary Materials. Data were extracted using SAS 7.1 (SAS Institute Inc., 2014) and analysed using Stata MP version 16.0 (StataCorp, 2019), and two-tailed α = 0.05 defined significance. To assess the model’s predictive power, the *c*-statistic was employed. This gives the probability a randomly selected participant who experienced an exposure (e.g., mental health indication) had a higher risk score than a participant who had not experienced the exposure. Model fit is typically considered reasonable when the c-statistic is higher than 0.7 and strong when the *c*-statistic exceeds 0.8^55^.

### Approvals and ethics

This study is a secondary analysis of routinely-collected de-identified administrative information housed and curated by Statistics New Zealand. The NZIDI is designed to be a research database that holds de-identified administrative records about people and households that come from government agencies, Statistics New Zealand surveys, and non-government organisations. Access to the data was provided by Statistics New Zealand under conditions designed to give effect to the security and confidentiality provisions of the Statistics Act 1975^38^. The data that support the findings of this study are available from Statistics New Zealand, but restrictions apply to the availability of these data which were used under license for the current study, and so are not publicly available. The data and code used for the purpose of the study are, however, available upon reasonable request from Statistics New Zealand. The data can only be accessed by approved bone fide researchers, for projects that are in the public interest and within a secure accredited data lab (see: https://www.stats.govt.nz/integrated-data/apply-to-use-microdata-for-research). Statistics New Zealand use the ‘Five Safes' and 'Nga Tikanga Paihere' frameworks to manage safe access to the information. Further description is given by: https://www.stats.govt.nz/integrated-data/integrated-data-infrastructure/your-information-in-the-idi/. The study proposal and protocols were approved by Statistics New Zealand (MAA2017-16). Based on New Zealand’s Health and Disability Ethics Committees’ (HDEC) checklist, the study did not meet the threshold required for formal ethics review. The University of Otago Human Research Ethics Committee reviewed the study for ethics consideration. The study was reviewed as a ‘Minimal Risk Health Research—Audit and Audit related studies’ proposal and was approved (reference: HD17/004). Informed consent from participants was not deemed necessary according to national legislation, i.e., the Statistics Act 1975^38^. All methods and reported results were carried out in accordance with relevant Statistics New Zealand and HDEC guidelines and regulations, and only includes aggregated randomly rounded to base 3 de-identified data. Random rounding to base 3 (RR3) involves randomly changing each count in a table to a multiple of 3. This is required by Statistics New Zealand in order to meet data confidentiality requirements.

## Results

### Participants

Over the 2013–2017 study baseline periods, 286,176 pupils who met the ERP criteria were enrolled in school Year 9. However, 9549 pupils were excluded as they were in another country during the 2-year mental health condition identification period, as were 3471 pupils who were in another country for at least 12 months during the follow-up period (school Years 9–11), and 255 who were aged under 12 years or over 15 years at the beginning of secondary school Year 9. This left a final analytical sample, who had data on prior mental health conditions and both educational outcomes, of n = 272,901 (95.4%).

### Sociodemographics

Table 1 reports the sociodemographic characteristics of the analytical sample. Of these, 133,584 (48.9%) were female, 74,481 (27.3%) and 36,354 (13.3%) identified themselves as Māori and/or Pacific peoples respectively, and the majority lived in urban residential locations (230,604, 84.5%).

**Table 1:** Sociodemographic distribution of the full sample (n = 272,901), and among those with any (n = 18,603, 6.8%), behavioural (n = 2,286, 0.8%), or emotional (n = 10,635, 3.9%) mental health condition.

	Full sample	Mental health condition		
		Any	Behavioural	Emotional
	n (%)	n (%)	n (%)	n (%)
Sex				
Female	133,584 (48.9)	8490 (45.6)	615 (26.9)	5187 (48.8)
Male	139,317 (51.1)	10,113 (54.4)	1671 (73.1)	5448 (51.2)
Ethnicity				
European/other	199,647 (73.2)	14,127 (75.9)	1845 (80.7)	8097 (76.1)
Māori	74,481 (27.3)	5640 (30.3)	750 (32.8)	2841 (26.7)
Pacific peoples	36,354 (13.3)	2310 (12.4)	282 (12.3)	1335 (12.6)
Asian	30,657 (11.2)	1521 (8.2)	99 (4.3)	1035 (9.7)
MELAA	3717 (1.4)	222 (1.2)	18 (0.8)	135 (1.3)
Deprivation				
1 (least deprived)	61,242 (22.4)	3717 (20.0)	357 (15.6)	2304 (21.7)
2	51,867 (19.0)	3405 (18.3)	378 (16.5)	2052 (19.3)
3	47,787 (17.5)	3369 (18.1)	408 (17.8)	1974 (18.6)
4	47,805 (17.5)	3453 (18.6)	498 (21.8)	1917 (18)
5 (most deprived)	60,090 (22.0)	4398 (23.6)	624 (27.3)	2238 (21)
Residential location				
Urban	230,604 (84.5)	15,774 (84.8)	1974 (86.4)	8988 (84.5)
Rural	38,493 (14.1)	2592 (13.9)	291 (12.7)	1518 (14.3)
Parents highest educational attainment				
Not reported	45,522 (16.7)	3381 (18.2)	504 (22.0)	1683 (15.8)
No qual	25,770 (9.4)	2067 (11.1)	321 (14.0)	1026 (9.6)
School qual	72,369 (26.5)	4935 (26.5)	609 (26.6)	2844 (26.7)
Post school qual	59,439 (21.8)	4122 (22.2)	450 (19.7)	2514 (23.6)
University qual	69,807 (25.6)	4101 (22)	402 (17.6)	2571 (24.2)
Household Income (NZD)				
Not reported	70,023 (25.7)	5046 (27.1)	675 (29.5)	2625 (24.7)
≤ $25,000	19,191 (7.0)	1536 (8.3)	225 (9.8)	777 (7.3)
$25,001–$50,000	33,891 (12.4)	2679 (14.4)	393 (17.2)	1458 (13.7)
$50,001–$100,000	74,802 (27.4)	5136 (27.6)	621 (27.2)	3108 (29.2)
$100,001–$150,000	41,706 (15.3)	2538 (13.6)	243 (10.6)	1608 (15.1)
> $150,000	33,291 (12.2)	1671 (9.0)	129 (5.6)	1062 (10.0)

Data source is the NZ-IDI. Counts and column percentages for ethnicity do not add to 100%, because the total response ethnicity coding is used where categories are not mutually exclusive—an individual may belong to more than one ethnic group; 1.5% of the sample were missing for deprivation; 1.4% were missing the urban/rural indicator; 0.01% were missing data for ethnicity.

### Identification of mental health conditions

Overall, 2286 (0.8%) participants were identified as having a behavioural mental health condition, 10,635 (3.9%) with an emotional mental health condition, and 18,603 (6.8%) participants with a mental health condition of any type. Table 1 also presents the distribution of sociodemographic variables for those with any, behavioural, or emotional conditions. It is evident from Table 1 that several patterns emerge, supporting the confounding relationships between mental health conditions and sociodemographic variables. For example, males are over-represented among those with a behavioural mental health condition (54.4% were male), whereas the sex split is less pronounced for emotional mental health conditions (51.2% were male). Social patterning is evident, particularly for area deprivation. Those living in more deprived areas are over-represented among those with behavioural conditions (27.7% resided in the most deprived quintile of areas), but less so for any mental health or emotional conditions (21.0% resided in the most deprived quintile of areas).

### Educational outcomes

Overall, by the end of Year 11, 205,638 (75.4%) of participants gained their NCEA Level 1 Certificate. However, over their Year 9–11 school tenure, 26,748 (9.8%) participants also had at least one period of stand-down or suspension. The sociodemographic distribution of the sample conditional on experiencing these educational outcomes, is presented in Table 2. Again, differential patterns emerge. While the proportion gaining NCEA Level 1 Certificate attainment was similar by sex (51.7% were female), females were under-represented among those being stood-down or suspended (34.6% were female). In contrast, Māori and Pacific peoples, and those living in more deprived areas, were under-represented among those with NCEA Level 1 Certificate attainment (among those gaining NCEA Level 1, 17.4% lived in the most deprived quintile of areas) and over-represented among those experiencing stand-down and suspension (among those who were stood-down or suspended, 40.9% lived in the most deprived quintile of areas).

**Table 2:** Sociodemographic distribution of the full sample (n = 272,901), and among those gaining NCEA Level 1 Certificate at Year 11 (n = 205,638, 75.4%), and among those being stood-down or suspended in school Years 9–11 (n = 26,748, 9.8%).

	Full sample	Educational outcomes	
		Gained NCEA Level 1	Stood-down/suspended
	n (%)	n (%)	n (%)
Sex			
Female	133,584 (48.9)	106,215 (51.7)	9258 (34.6)
Male	139,317 (51.1)	99,423 (48.3)	17,490 (65.4)
Ethnicity			
European/other	199,647 (73.2)	158,520 (77.1)	15,852 (59.3)
Māori	74,481 (27.3)	46,293 (22.5)	13,578 (50.8)
Pacific peoples	36,354 (13.3)	23,817 (11.6)	5571 (20.8)
Asian	30,657 (11.2)	24,516 (11.9)	1113 (4.2)
MELAA	3717 (1.4)	2865 (1.4)	291 (1.1)
Deprivation			
1 (least deprived)	61,242 (22.4)	52,614 (25.6)	2547 (9.5)
2	51,867 (19.0)	42,525 (20.7)	3186 (11.9)
3	47,787 (17.5)	37,212 (18.1)	4065 (15.2)
4	47,805 (17.5)	34,206 (16.6)	5688 (21.3)
5 (most deprived)	60,090 (22.0)	35,724 (17.4)	10,932 (40.9)
Residential location			
Urban	230,604 (84.5)	172,035 (83.7)	22,899 (85.6)
Rural	38,493 (14.1)	30,480 (14.8)	3555 (13.3)
Parents highest educational attainment			
Not reported	45,522 (16.7)	28,365 (13.8)	7626 (28.5)
No qual	25,770 (9.4)	14,007 (6.8)	5226 (19.5)
School qual	72,369 (26.5)	54,225 (26.4)	7266 (27.2)
Post school qual	59,439 (21.8)	48,681 (23.7)	4155 (15.5)
University qual	69,807 (25.6)	60,366 (29.4)	2475 (9.3)
Household Income (NZD)			
Not reported	70,023 (25.7)	45,312 (22.0)	10,818 (40.4)
≤ $25,000	19,191 (7.0)	11,880 (5.8)	3057 (11.4)
$25,001–$50,000	33,891 (12.4)	23,706 (11.5)	4209 (15.7)
$50,001–$100,000	74,802 (27.4)	59,904 (29.1)	5616 (21.0)
$100,001–$150,000	41,706 (15.3)	35,844 (17.4)	1950 (7.3)
> $150,000	33,291 (12.2)	28,995 (14.1)	1095 (4.1)

Data source is the NZ-IDI. Counts and column percentages for ethnicity do not add to 100%, because the total response ethnicity coding is used where categories are not mutually exclusive—an individual may belong to more than one ethnic group; 1.5% of the sample were missing for deprivation; 1.4% were missing the urban/rural indicator; 0.01% were missing data for ethnicity.

### Cross-tabulations of mental health condition and educational outcomes

Table 3 presents the percentage experiencing each educational outcome for the full sample, as well as separately for those experiencing each mental health condition. Among those with any mental health condition 65% gained a NCEA Level 1 Certificate compared to 76.1% among those without, hence the majority of those with any mental health condition still achieved the certificate. This difference was observed for both boys and girls, although girls have higher rates of gaining NCEA Level 1 compared with boys (see Supplementary Materials, Table A2). Similarly, among those with any mental health condition 16.2% were stood-down or suspended, compared to 9.3% among those with no mental health condition, hence the overwhelming majority of those with a mental health indication were neither stood-down nor suspended. This difference was also observed among boys and girls, with a larger difference for boys (21.0% were suspended among boys with any mental health condition, compared to 11.9% of boys with no mental health condition, the figures for girls being 10.6% and 6.7% respectively; see Supplementary Materials, Table A2).

**Table 3:** Percentages experiencing each educational outcome for the full sample (n = 272,901) and separately by mental health condition.

Mental health condition	N	Educational outcomes	
		Gained NCEA Level 1	Stood-down/suspended
		n (%)	n (%)
Full sample	272,901	205,638 (75.4)	26,748 (9.8)
Any mental health condition			
Yes	18,603	12,099 (65.0)	3015 (16.2)
No	254,298	193,539 (76.1)	23,733 (9.3)
Behavioural condition			
Yes	2286	957 (41.9)	741 (32.4)
No	270,615	204,681 (75.6)	26,007 (9.6)
Emotional condition			
Yes	10,635	7737 (72.8)	1149 (10.8)
No	262,266	197,901 (75.5)	25,599 (9.8)

Data source is the NZ-IDI.

When examining behavioural and emotional mental health conditions separately, stand-down and suspension rates were higher (32.4%), and NCEA Level 1 Certificate attainment lower (41.9%), among those experiencing behavioural conditions compared to the full population (9.8% and 75.4%, respectively), whereas those experiencing emotional health conditions had more similar outcomes to the full study population (10.8% and 72.8%, respectively); see Table 3. These patterns were consistent among both boys and girls (Supplementary Materials, Table A2). However, the extent to which these patterns may be explained by confounding from sociodemographic characteristics observed in Tables 1 and 2, behoves the pursuant adjusted analyses.

### Adjusted analyses

Table 4 reports the results from assessing the relationship between education outcomes and mental health conditions using this regression framework. Model 0 adjusted only for study cohort indicators, and showed that those experiencing a prior mental health condition had a reduced probability of gaining a NCEA Level 1 Certificate by a factor of 0.87 (95% confidence interval [CI] 0.86, 0.88). This estimate remained largely unchanged after adjusting for sociodemographic variables. The IRR was 0.86 in the fully adjusted model (95% CI 0.84, 0.88) among boys and 0.92 (0.90, 0.93) among girls (see Supplementary Materials, Table A2). Examining emotional and behavioural conditions separately, behavioural conditions were associated with a markedly larger reduction in the probability of gaining a NCEA Level 1 Certificate. In Model 0, stand-down and/or suspension rates were 1.63 (95% CI 1.57, 1.70) times higher among those with any mental health condition. This figure is attenuated to 1.50 (95% CI 1.50, 1.55) after adjustment for sociodemographic covariates. IRRs were higher among boys (1.54 (95% CI 1.48, 1.60)) compared with girls (1.13 (95% CI 1.04, 1.24)). This association appeared to be largely driven by those with behavioural conditions, for whom the rate of stand-downs and/or suspensions was 2.84 (95% CI 2.63, 3.07) times higher. After adjusting for covariates, this figure remained high at 2.31 (95% CI 2.14, 2.49), and was similar for both boys and girls (Supplementary Materials, Table A2). In contrast, the estimates associated with emotional conditions were 1.09 (95% CI 1.04, 1.16) in the unadjusted model and 1.10 (95% CI 1.04, 1.16) after full adjustment for covariates; the fully adjusted figures were 1.09 (95% CI 1.10, 1.16) among boys, and 1.43 (95% CI 1.34, 1.53) among girls (Supplementary Materials, Table A2). The *c*-statistic was largest in the fully adjusted model, with values ranging from 0.72 to 0.76 demonstrating reasonable predictive power.

**Table 4:** Incidence rate ratios (IRR), with associated and 95% confidence intervals (CIs), for the relationship between mental health conditions and gaining NCEA Level 1 Certificate at Year 11 and stand-down/suspensions, respectively.

	Model 0	Model 1	Model 2
	IRR (95% CI)	IRR (95% CI)	IRR (95% CI)
	N = 272,901	N = 272,862	N = 268,761
NCEA—Level 1 certificate			
Any mental health condition			
Yes	0.87 (0.86, 0.88)	0.88 (0.87, 0.89)	0.89 (0.88, 0.90)
No	1 (reference)	1 (reference)	1 (reference)
* c*-statistic	0.54	0.67	0.72
Behavioural condition			
Yes	0.59 (0.56, 0.62)	0.60 (0.57, 0.64)	0.62 (0.59, 0.65)
No	1 (reference)	1 (reference)	1 (reference)
* c*-statistic	0.54	0.67	0.72
Emotional condition			
Yes	0.97 (0.96, 0.98)	0.97 (0.96, 0.98)	0.97 (0.96, 0.98)
No	1 (reference)	1 (reference)	1 (reference)
* c*-statistic	0.53	0.67	0.71
Stand-downs and/or suspensions			
Any mental health condition			
Yes	1.63 (1.57, 1.70)	1.56 (1.51, 1.62)	1.50 (1.50, 1.55)
No	1 (reference)	1 (reference)	1 (reference)
* c*-statistic	0.54	0.71	0.76
Behavioural condition			
Yes	2.84 (2.63, 3.07)	2.51 (2.33, 2.72)	2.31 (2.14, 2.49)
No	1 (reference)	1 (reference)	1 (reference)
* c*-statistic	0.53	0.71	0.76
Emotional condition			
Yes	1.09 (1.04, 1.16)	1.10 (1.05, 1.16)	1.10 (1.04, 1.16)
No	1 (reference)	1 (reference)	1 (reference)
* c*-statistic	0.52	0.71	0.76

Data source is the NZ-IDI. Model 0 included indicators for school cohort; Model 1 included indicators for school cohort, child’s sex, age-at-school-entry, ethnicity; Model 2 included indicators for school cohort, child’s sex, age-at-school-entry, ethnicity, highest parental education level, household income bands, quintiles of area-deprivation and residential location. *c*-statistic is Harrell’s c-statistic. The complete-case samples for Models 1 and 2 were lower than the full sample in Model 0, due to 1.5% of the sample being missing for deprivation; 1.4% missing the urban/rural indicator; 0.01% missing data for ethnicity.

## Discussion

Using a near-complete population of New Zealand young people entering secondary school during the period 2013–2017, prevalence of mental health condition (6.8% for any type) was significantly associated with lower rates of attainment and higher rates of stand-downs and/or suspensions by school Year 11. These relationships endured after adjusting for a suite of sociodemographic factors. These findings are in line with a literature which studies links between mental health and educational outcomes^24–26,56^, across a range of data types and study designs^27,28^. This study provides timely evidence for the New Zealand context, where inequalities in mental health and education have been documented^6,21^, but there is less evidence studying the connection between these two important variables. The observed associations were especially pronounced among those young people exhibiting behavioural conditions, compared to emotional conditions. Previous studies have also documented negative outcomes associated with externalising conditions, such as ADHD, and highlighted a need for greater awareness of these conditions and additional support for schools in supporting these students^57^. The smaller association between emotional conditions may be due to heterogeneity within this category, as outcomes for those with mental health conditions are likely to vary depending on the specific type of condition, its symptoms and severity. Anxiety, for instance, can manifest via a diverse range of symptoms which may each associate differently with educational outcomes. Differential associations by type of mental health condition have been identified in previous literature, for instance, a recent study found that obsessive–compulsive disorder and anorexia nervosa were associated with higher grades in secondary school^29^. Further work examining outcomes associated with specific mental health conditions may shed light on heterogeneity in outcomes for young people with mental health conditions.

While measured effect sizes were at times large, and generally poorer for those identified as having a mental health condition, it must be emphasised that the vast majority of children gained a NCEA Level 1 Certificate and did not experience stand-downs or suspensions—regardless of their mental health status. Indeed, of those with any mental health condition, 65.0% attained NCEA Level 1 Certificate and 83.8% never experienced a stand-down or suspension. Poor academic outcomes, as measured here using two sentinel indices, do not necessarily result from having a mental health condition. Māori and Pacific peoples were also under-represented among those gaining a NCEA Level 1 Certificate attainment, and over-represented among those experiencing stand-down and suspension. Evidently there are inequities occurring for Māori and Pacific young people, however it is outside the scope of this study to discuss the socio-political experience of these groups. The investigation of the underlying determinants and potential solutions are best addressed from within those communities.

Methodologically, this study highlights the utility of administrative data for studying links between health and education. While sample survey data have many advantages, including the richness of variables collected, they also have potential to suffer from high rates of non-response, smaller sample sizes, recall and self-reporting bias, and can be relatively costly to collect and to maintain high response rates. For example, English Avon Longitudinal Study of Parents and Children birth cohort study (ALSPAC) cost an estimated £1 million per year to follow 14,000 families over 20 years^58,59^. The use of linked, routinely-collected records, may offer a lower-cost complement to sample-surveys and birth cohort studies to inform on the aetiology and consequences of mental health in childhood and adolescence. They also enable the development of contemporary, virtual or synthetic investigator defined cohorts and data which purposefully address the research question at hand^60^.

### Strengths and limitations

The use of linked administrative data is eminently suitable for contributing to the health and education nexus^61^, and the NZIDI in particular has several attractive properties for this study. First, the large sample sizes facilitate analyses of infrequent events (such as stand-downs and suspensions) and subgroup analyses. Second, the use of administrative records removes concern of differential self-report bias which is common in sample-surveys. Additionally, compared to other studies in this area, which typically use either service-use alone or an ICD code diagnosis^29^, we used a novel classification method for identifying probable mental health conditions among young people which combines information from use of multiple services, diagnostic codes and clinical judgement^37^. However, the study is not without limitations. While the employed mental health case classification approach overcomes several important concerns in estimating the prevalence of mental health conditions, the method does rely on secondary service use data, among which severe mental health conditions may be over-represented. Similarly, there may be undercounting of cases for those who do not seek medical care. The extent of any undercounting or false positive rate is currently unknown. Investigating this, potentially via means of comparison with analogous survey data, represents a fruitful direction for further research. Additionally, because we use official records of suspensions and stand-downs, any informal encouragement to keep a child away from school due to behavioural problems would not be captured in our data, potentially underestimating the true extent of suspensions^62^.

Further, as while this is a large prospective study, its observational nature, together with the likely unbalanced confounder pathways and patterns (both known and unknown) means that it is difficult to assert causality^63^. For example, early-life circumstance may impact on the probability of developing a mental health condition as well as shaping educational outcomes and attainment, thus generating a spurious correlation between these two variables. However, a comprehensive set of sociodemographic variables was used to gauge the confounding effects and attempt to mitigate this impact. Moreover, a link between mental health conditions and educational outcomes has also been observed across studies using alternative study designs which rely on different assumptions, such as Mendelian randomisation^27,28^, and family fixed effect models^9^, which provides supporting evidence for the veracity of this association. Still, even if these differences are not causal effects—that is, only due to mental health—or indeed more likely, some mix of correlation and causation, it remains important to document health-related inequalities in educational outcomes. Gaps in educational attainment highlight that there are young people with support needs which are not being met by the current system, and who are experiencing poorer outcomes at an important juncture in their school career. This was highlighted in a recent study of young people in New Zealand, which showed autistic students experienced significantly higher odds of suspension compared with their non-autistic peers, but this was mitigated by the receipt of high-need education-based funding^64^. Although those findings were for Autism Spectrum Disorder, rather than the mental health conditions considered in this paper, it highlights the potential of in-school support and funding as a means of reducing health-related educational inequalities.

Finally, in this study, differences between two groups were identified at one particular—and important—moment of early adolescence. However, the links between education and mental health are complex, dynamic and bi-directional, and this study has not elucidated the precise mechanisms through which mental health may impact on later outcomes, or indeed the extent to suspensions may explain mental health’s association with attainment. This represents an avenue for further work. The external validity of the findings will also be impacted by the COVID-19 crisis. In the post COVID-19 era, both mental health and educational outcome landscapes will likely have been altered in the medium-term, if not irrevocably. Only future studies conducted in this era will illuminate the extent of this impact, and inform on how policy and practice may need to change to accommodate the consequences of COVID-19.

## Conclusions

Studying a near-complete population of New Zealand young people entering secondary school during the period 2013–2017, prevalence of mental health condition was significantly associated with lower rates of attainment and higher rates of stand-downs and/or suspensions by age 15–16 years. While mental health conditions increase the risk of poorer educational outcomes, deleterious outcomes are, however, not a necessary sequalae; here, most participants with mental health conditions had successful educational outcomes. Future investigations which explore and understand the different positive trajectories of adolescences with mental health conditions may provide pathways to reduce the educational attainment differences observed here and elsewhere.

## Supplementary Information

Supplementary Information.

## Data Availability

The datasets used for statistical analysis are held by Statistics New Zealand. These results are not official statistics. They have been created for research purposes from the Integrated Data Infrastructure (IDI) which is carefully managed by Stats NZ. For more information about the IDI please visit https://www.stats.govt.nz/integrated-data/. The data that support the findings of this study are available from Statistics New Zealand, but restrictions apply to the availability of these data, which were used under license for the current study, and so are not publicly available. The data and code used in this study are however available from the authors upon reasonable request and with permission of Statistics New Zealand (see: https://www.stats.govt.nz/integrated-data/apply-to-use-microdata-for-research). The data can only be accessed by approved bone fide researchers, for projects that are in the public interest and within a secure accredited data lab.
