# Supplementary material for: A national multiple baseline cohort study of mental health conditions in early adolescence and subsequent educational outcomes in New Zealand
Source: Sci Rep. 2023 Jul 7;13:11025. doi: 10.1038/s41598-023-38131-8 (PMC10329034; doi:10.1038/s41598-023-38131-8)
Supplement: Supplementary file 1 — Supplementary Information. [file 41598_2023_38131_MOESM1_ESM.docx]

**Supplementary Materials**

**Table A1** Codes used in the mental health classification methodology

| **Mental Health Problem Group** | **NMDS (ICD-10-AM)** | **PRIMHD (DSM-IV plus ICD-10-AM codes listed under NMDS)** | **PRIMHD Team type / Activity type** | **Pharmaceuticals** | **Socrates** |
| --- | --- | --- | --- | --- | --- |
| Anxiety | ICD-10-AM = F411, F4000^b^, F401^a^, F402, F408, F409, F931, F4001^b^, F410^b^, F412, F413, F418, F419, F064, F932, F420^a^, F421^a^, F422^a^, F428^a^,F429^a^, F450, F451, F452, F4530, F4531, F4532, F4533, F4534, F4535, F4538, F4539, F454, F458, F459, F480, F680, F681, F930, F430, F431, F432, F438, F439 | DSM-IV = 30,002, 30,029, 30,022^b^, 30023^a^, 30,001^b^, 30,021^b^, 30,000, 29,384, 31,323, 3003^a^, 3007, 30,081, 30,082, 30,780, 30,789, 30,016, 30,019, 30,921, 3083, 30,981, 30,924, 30,928 | None | Chemical ID = 6006, 1166, 1780, 2632, 1911, 1080^c^, 1730^b^, 1316^b^, 2636 (age 5–9 only) | Assigned diagnosis code = 1302 |
| Depression | ICD-10-AM = F320^a^, F3200^a^, F3201%, F321^a^, F3210^a^, F3211%, F322^a^, F3220^a^, F3221%, F323^a^, F3230^a^, F3231%, F328^a^, F3280^a^, F3281%, F329^a^, F3290^a^, F3291%, F330%, F331%, F332%, F333%, F334%, F338%, F339%, F341%, F348%, F349%, F380^a^, F381%, F388^a^, F39^a^, F412, F251^a^, F0633 | DSM-IV = 30,928, 29,620^a^, 29621^a^, 29622^a^, 29623^a^, 29624^a^, 29625^a^, 29626^a^, 29,630, 29,631, 29,632, 29,633, 29,634, 29,635, 29,636^c^, 29690^a^, 3004^a^, 3090^a^, 311^a^ | None | Chemical ID = 1437, 1438, 3753, 1824, 2285, 2301, 3901, 1760, 2638, 1180, 3785, 1379^c^, 1642^d^, 1059^e^ | Assigned diagnosis code = 1304 |
| Emotional Problems | ICD-10-AM = F938, F939, F928, F929, F252^b^, F258^b^, F063, F0630, F0634, F0639, F920 | DSM-IV = 3094, 3099, 29,383 | None | Chemical ID = 2636^b^, 3926^b^, 3927^b^, 1030^b^, 1193^b^, 1190^b^, 1955, 6009, 1069, 1125^c^ | None |
| Bipolar Disorders^c^ | ICD-10-AM = F310, F311, F312, F313, F314, F315, F316, F317, F318, F319, F300, F301, F302, F308, F309, F340, F0631, F0632 | DSM-IV = 29,600, 29,601, 29,602, 29,603, 29,604, 29,605, 29,606, 29,640, 29,641, 29,642, 29,643, 29,644, 29,645, 29,646, 29,650, 29,651, 29,652, 29,653, 29,654, 29,655, 29,656, 29,660, 29,661, 29,662, 29,663, 29,664, 29,665, 29,666, 2967, 29,680, 29,689, 30,113 | None | None | Assigned diagnosis code = 1303 |
| Substance Problems^b^ | ICD-10-AM = F100, F101, F102, F103, F104, F105, F106, F107, F108, F109, F110, F111, F112, F113, F114, F115, F116, F117, F118, F119, F120, F121, F122, F123, F124, F125, F126, F127, F128, F129, F130, F131, F132, F133, F134, F135, F136, F137, F138, F139, F140, F141, F142, F143, F144, F145, F146, F147, F148, F149, F150, F151, F152, F153, F154, F155, F156, F157, F158, F159, F160, F161, F162, F163, F164, F165, F166, F167, F168, F169, F180, F181, F182, F183, F184, F185, F186, F187, F188, F189, F190, F191, F192, F193, F194, F195, F196, F197, F198, F199, F550, F551, F552, F553, F554, F555, F556, F558, F559 | DSM-IV = 30,300, 30,500,30,390, 2910, 2913, 2915, 29,181, 29,189, 2919, 30,430, 30,520, 30,400, 30,550, 30,410, 30,540, 30,480, 30,490, 30,420, 30,560, 30,450, 30,530, 30,460, 30,590, 2920, 29,211, 29,212, 29,281, 29,284, 29,289, 2929, 30,440, 30,570 | Activity type = T16, T17, T18, T19, T20, and/or Team type = 3 | Chemical ID = 2367, 1432, 1841, 3950, 1795, 3793, 1252, 1273 | Assigned diagnosis code = 1301 |
| Eating Problems | ICD-10-AM = F500^b^, F501^b^, F502^b^, F503^b^, F508, F509, F982, F983 | DSM-IV = 3071^b^, 30750^a^, 30,751^b^, 30,752, 30,753, 30,759 | Team type = 16 | None | None |
| Disruptive Behaviours | ICD-10-AM = F900, F908, F909, F901, F910, F911, F912, F918, F919, F920, F928, F929, F913, F631, F632, F638, F639 | DSM-IV = 31,400, 31,401, 3149, 3093, 3094, 31,281, 31,282, 31,289, 3129, 31,381, 31,230, 31,232, 31,233, 31,234, V7102 | None | Chemical ID = 3887^a^, 1809, 3880, 1389 | Assigned diagnosis code = 1201, 1307 |
| Psychosis^b^ | ICD-10-AM = F200, F201, F202, F203, F204, F205, F206, F208, F209, F21, F220, F228, F229, F230, F231, F232, F233, F238, F239, F24, F250, F251, F252, F258, F259, F28, F29, F105, F115, F125, F135, F145, F155, F165, F175, F185, F195 | DSM-IV = 29,510, 29,520, 29,530, 29,540, 29,560, 29,570, 29,590, 2971, 2973, 2988, 2989, 2913, 2915, 29,211, 29,212, 29,381, 29,382 | Activity type = T09 | Chemical ID = 3884, 1078, 1532, 2820, 1732, 1990, 1994, 2255, 2260, 1533, 1535, 1950, 3873,1007, 1226, 1283, 1583, 1799, 2298, 2530, 3803, 3898,3940, 4025, 8792 | Assigned diagnosis code = 1306 |
| Personality Disorders^e^ | ICD-10-AM = F600, F601, F602, F6030, F6031, F604, F605, F606, F607, F608, F609, F61, F620, F621, F628, F629, F070 | DSM-IV = 3010, 3019, 30,120, 30,122, 3014, 30,150, 3016, 3017, 30,181, 30,182, 30,183 | None | None | None |
| Sleep Problems | ICD-10-AM = F510, F511, F512, F518, F519, F513, F514, F515 | DSM-IV = 30,742, 30,744, 30,745, 347, 78,052, 78,059, 30,746, 30,747 | None | Chemical ID = 3735, 2484^c^ | None |
| Self-harm (self-harm also uses the ICD-10-AM codes listed under NMDS within mortality collection data) | ICD-10-AM = X60, X61, X62, X63, X64, X65, X66, X67, X68, X69, X70, X71, X72, X73, X74, X75, X76, X77, X78, X79, X80, X81, X82, X83, X84, Y870 | None | None | None | None |
| Other Mental Health | ICD-10-AM = F050, F051, F058, F059, F060, F061, F062, F065, F066, F068, F069, F071, F072, F078, F079, F09, F488, F489, F633, F842, F843, F950, F951, F952, F958, F959, F980, F981, F984, F988, F989, F99, F630^b^ | DSM-IV = 2930, 29,389, 2939, 29,910, 30,720, 30,721, 30,722, 30,723, 3073, 3076, 3077, 31,239, 31,382, 31,389, 3139, 78,009, 7876, 31,231^b^ | None | None | None |
| Mental health not defined | None | DSM-IV = V7109, 3009, 7999 | None | Chemical ID = 2466^c^, 3878^a^, 1315^a^, 1140^a^, 1183^a^, 1011^a^, 1729, 1731, 2295, 1397, 1865, 2224, 2436, 3892, 6007^c^ | Assigned diagnosis code = 1399 |

*Notes:* This table reports the codes used to classify mental health conditions as developed in: Bowden, N. *et al.* Case identification of mental health and related problems in children and young people using the New Zealand Integrated Data Infrastructure. *BMC Med. Inform. Decis. Mak.* **20**, 1–13. https://doi.org/10.1186/s12911-020-1057-8 (2020). Team Type codes describe the specialist area of teams who provide a mental health service, and Activity Type codes categorise the health care activities provided by each team.

a Restricted to ages 5+

b Restricted to ages 10+

c Restricted to ages 15+

d Restricted to ages 20+

e Restricted to ages 18+

**Table A2.** Percentages experiencing each educational outcome for boys and girls, and separately by mental health condition

|  |  |  | Boys (N=139,314) | |  | Girls (N=133,587) |  |  |  |
| --- | --- | --- | --- | --- | --- | --- | --- | --- | --- |
| Mental health condition | |  | Gained NCEA Level 1 | Stood-down/suspended | N | Gained NCEA Level 1 | Stood-down/suspended |  |  |
|  | | N | n (%) | n (%) |  | n (%) | n (%) |  |  |
| *Full sample* | | 139314 | 99423 (71.4) | 17493 (12.6) | 133587 | 106218 (79.5) | 9258 (6.9) |  |  |
| *Any mental health condition* | | |  |  |  |  |  |  |  |
|  | Yes | 10116 | 6021 (59.5) | 2121 (21) | 8490 | 6072 (71.5) | 900 (10.6) |  |  |
|  | No | 129198 | 93402 (72.3) | 15372 (11.9) | 125097 | 100146 (80.1) | 8358 (6.7) |  |  |
| *Behavioural condition* | | |  |  |  |  |  |  |  |
|  | Yes | 1671 | 624 (37.3) | 615 (36.8) | 618 | 339 (54.9) | 120 (19.4) |  |  |
|  | No | 127527 | 98799 (77.5) | 16878 (13.2) | 124479 | 105879 (85.1) | 9138 (7.3) |  |  |
| *Emotional condition* | | |  |  |  |  |  |  |  |
|  | Yes | 5445 | 3741 (68.7) | 744 (13.7) | 5190 | 3990 (76.9) | 399 (7.7) |  |  |
|  | No | 122082 | 95682 (78.4) | 16749 (13.7) | 119289 | 102228 (85.7) | 8859 (7.4) |  |  |

Notes: Data source is the NZ-IDI.

**Table A3.** Incidence rate ratios (IRR), with associated and 95% confidence intervals (CIs), for the relationship between mental health conditions and gaining NCEA Level 1 Certificate at Year 11 and stand-down/suspensions, respectively, stratified by sex.

|  | | **Boys** | | | **Girls** | | |
| --- | --- | --- | --- | --- | --- | --- | --- |
|  | | Model 0 | Model 1 | Model 2 | Model 0 | Model 1 | Model 2 |
|  | | IRR (95% CI) | IRR (95% CI) | IRR (95% CI) | IRR (95% CI) | IRR (95% CI) | IRR (95% CI) |
| **NCEA – Level 1 Certificate** | | N=139,317 | N=139,293 | N=137,109 | N=133,584 | N=133,569 | N=131,655 |
| *Any mental health condition* | | |  |  |  |  |  |
|  | Yes | 0.843 (0.826, 0.86) | 0.847 (0.83, 0.864) | 0.860 (0.844, 0.876) | 0.907 (0.893, 0.92) | 0.911 (0.898, 0.924) | 0.915 (0.902, 0.928) |
|  | No | 1 (reference) | 1 (reference) | 1 (reference) | 1 (reference) | 1 (reference) | 1 (reference) |
|  | *c*-statistic | 0.54 | 0.64 | 0.69 | 0.54 | 0.67 | 0.73 |
| *Behavioural condition* | | |  |  |  |  |  |
|  | Yes | 0.550 (0.517, 0.585) | 0.553 (0.519, 0.589) | 0.573 (0.538, 0.609) | 0.712 (0.661, 0.766) | 0.717 (0.667, 0.772) | 0.729 (0.679, 0.784) |
|  | No | 1 (reference) | 1 (reference) | 1 (reference) | 1 (reference) | 1 (reference) | 1 (reference) |
|  | *c*-statistic | 0.54 | 0.64 | 0.69 | 0.54 | 0.67 | 0.73 |
| *Emotional condition* | | |  |  |  |  |  |
|  | Yes | 0.966 (0.948, 0.984) | 0.963 (0.945, 0.981) | 0.967 (0.949, 0.985) | 0.974 (0.96, 0.988) | 0.972 (0.959, 0.986) | 0.972 (0.959, 0.985) |
|  | No | 1 (reference) | 1 (reference) | 1 (reference) | 1 (reference) | 1 (reference) | 1 (reference) |
|  | *c*-statistic | 0.53 | 0.64 | 0.69 | 0.53 | 0.67 | 0.73 |
| **Stand-downs and/or suspensions** | | | | |  |  |  |
| *Any mental health condition* | | |  |  |  |  |  |
|  | Yes | 1.646 (1.579, 1.716) | 1.611 (1.547, 1.678) | 1.541 (1.482, 1.603) | 1.121 (1.022, 1.229) | 1.137 (1.039, 1.244) | 1.134 (1.039, 1.236) |
|  | No | 1 (reference) | 1 (reference) | 1 (reference) | 1 (reference) | 1 (reference) | 1 (reference) |
|  | *c*-statistic | 0.54 | 0.68 | 0.73 | 0.54 | 0.72 | 0.77 |
| *Behavioural condition* | | |  |  |  |  |  |
|  | Yes | 2.601 (2.395, 2.825) | 2.555 (2.358, 2.767) | 2.337 (2.162, 2.527) | 2.575 (2.167, 3.06) | 2.566 (2.157, 3.052) | 2.377 (2.018, 2.8) |
|  | No | 1 (reference) | 1 (reference) | 1 (reference) | 1 (reference) | 1 (reference) | 1 (reference) |
|  | *c*-statistic | 0.52 | 0.68 | 0.73 | 0.53 | 0.72 | 0.77 |
| *Emotional condition* | | |  |  |  |  |  |
|  | Yes | 1.081 (1.008, 1.16) | 1.093 (1.021, 1.169) | 1.089 (1.019, 1.163) | 1.524 (1.424, 1.63) | 1.48 (1.385, 1.58) | 1.432 (1.344, 1.526) |
|  | No | 1 (reference) | 1 (reference) | 1 (reference) | 1 (reference) | 1 (reference) | 1 (reference) |
|  | *c*-statistic | 0.51 | 0.67 | 0.72 | 0.52 | 0.72 | 0.77 |

*Notes:* Data source is the NZ-IDI. Model 0 included indicators for school cohort; Model 1 included indicators for school cohort, child’s sex, age-at-school-entry, ethnicity; Model 2 included indicators for school cohort, child’s sex, age-at-school-entry, ethnicity, highest parental education level, household income bands, quintiles of area-deprivation and residential location. *c*-statistic is Harrell’s c-statistic. The complete-case samples for Models 1 and 2 were lower than the full sample in Model 0, due to 1.5% of the sample being missing for deprivation; 1.4% missing the urban/rural indicator; 0.01% missing data for ethnicity.
